# Ultrasensitive Aptasensors for the Detection of Viruses Based on Opto-Electrochemical Readout Systems

**DOI:** 10.3390/bios12020081

**Published:** 2022-01-29

**Authors:** Divya, Daphika S Dkhar, Rohini Kumari, Supratim Mahapatra, Rahul Kumar, Pranjal Chandra

**Affiliations:** Laboratory of Bio-Physio Sensors and Nanobioengineering, School of Biochemical Engineering, Indian Institute of Technology (BHU) Varanasi, Varanasi 221005, Uttar Pradesh, India; divya.rs.bce20@itbhu.ac.in (D.); daphikasdkhar.rs.bce21@itbhu.ac.in (D.S.D.); rohinikumari.rs.bce21@itbhu.ac.in (R.K.); supratimmahapatra.rs.bce20@itbhu.ac.in (S.M.); rahulk.rs.bce16@itbhu.ac.in (R.K.)

**Keywords:** aptamers, viral infection, COVID-19, human health, digital health, biosensor

## Abstract

Viral infections are becoming the foremost driver of morbidity, mortality and economic loss all around the world. Treatment for diseases associated to some deadly viruses are challenging tasks, due to lack of infrastructure, finance and availability of rapid, accurate and easy-to-use detection methods or devices. The emergence of biosensors has proven to be a success in the field of diagnosis to overcome the challenges associated with traditional methods. Furthermore, the incorporation of aptamers as bio-recognition elements in the design of biosensors has paved a way towards rapid, cost-effective, and specific detection devices which are insensitive to changes in the environment. In the last decade, aptamers have emerged to be suitable and efficient biorecognition elements for the detection of different kinds of analytes, such as metal ions, small and macro molecules, and even cells. The signal generation in the detection process depends on different parameters; one such parameter is whether the labelled molecule is incorporated or not for monitoring the sensing process. Based on the labelling, biosensors are classified as label or label-free; both have their significant advantages and disadvantages. Here, we have primarily reviewed the advantages for using aptamers in the transduction system of sensing devices. Furthermore, the labelled and label-free opto-electrochemical aptasensors for the detection of various kinds of viruses have been discussed. Moreover, numerous globally developed aptasensors for the sensing of different types of viruses have been illustrated and explained in tabulated form.

## 1. Introduction

**Figure 1:**
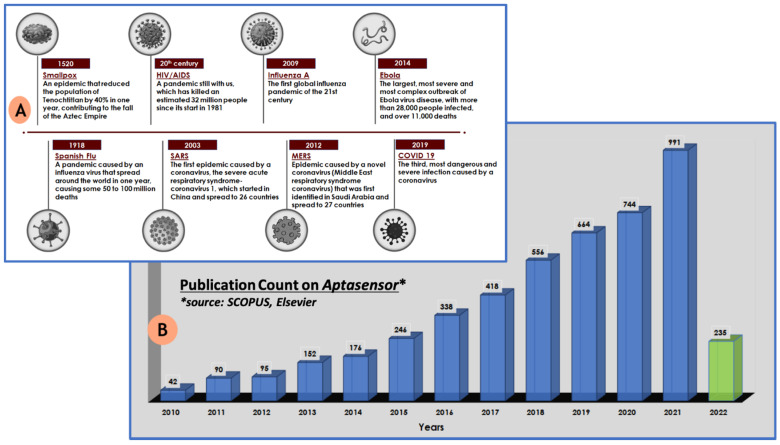
(**A**) Timeline of the major virus outbreaks that have occurred in past centuries that have created havoc to mankind; (**B**) graphical representation of the Scopus, Elsevier survey representing the growing interest towards aptasensors in the last decade.

**Figure 2:**
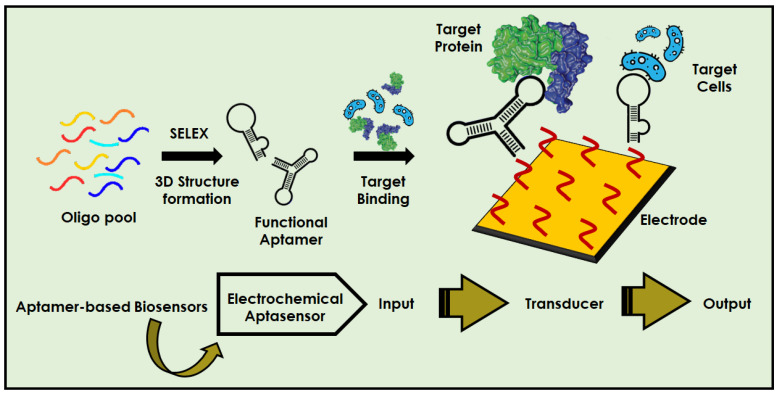
Pictorial representation of step-by-step fabrication procedure of aptasensors for the detection of various target molecules.

According to the World Health Organization, hundreds of millions of people have viral infections every year, with millions of death worldwide [1]. Figure 1A illustrates the the outbreak of few major viral infections that have created havoc in the human population over the past few centuries. The current situation of the COVID-19 pandemic has brought scientist’s focus towards infectious diseases caused by viruses. The ongoing pandemic by novel SARS-CoV-2 has reported more than 284 million infections and claimed around 5.4 million deaths [2]. In addition to this, various other viruses, such as HIV, Dengue, Zika, Ebola, West Nile, etc., lead to a large number of human infections and deaths. Therefore, simple, fast, accurate, and specific diagnostic techniques are in great demand for the early diagnosis, prognosis and surveillance of diseases, by targeting particular biomarkers/target sites. There are some reliable techniques which exist for virus detection such as cell culture, hemagglutination inhibition test, immuno-complexation, etc [3,4]. Furthermore, with advancement in technology, a few other techniques, for instance, ELISA, RIA, serological and PCR methods, have also been employed for virus detection [5,6]. However, these methods are costly, time consuming, and require laboratory infrastructure and skilled personnel. In contrast to these methods, point-of-care (POC) diagnostic tools have attracted more attention for their rapid, easy, and inexpensive detection methods, especially in the developing nations, which lacks proper infrastructure and trained individuals [7,8,9]. Additionally, owing to their excellent properties, POC devices have become very significant in the prevention and control of various viral infections [10,11,12,13]. Aptasensors are one of the most advanced diagnostic techniques, exhibiting excellent properties and great potential for the detection of viruses, especially in resource-constrained areas [14]. Aptamers were discovered in 1990, and since then they have been extensively used as molecular recognition elements in a large number of applications [15,16,17]. This is because they can probe for different targets, for example metal ions, small and macro molecules, and even cells with high selectivity and sensitivity. Biosensors are the analytical devices which modulates the signal obtained from the interaction between the biorecognition element (BRE) and the target molecule [18,19]. When aptamers are used as a BRE in biosensors to track a specific target or biomarker, an aptasensor is formed. The basic principle of designing and functioning of aptasensor, starting from the specific aptamer selection to signal generation, is represented in Figure 2. The signal obtained in the sensor can be in the form of a colour change, fluorescence, electrochemical parameters, mechanical parameters, etc [20,21,22,23,24].

This review mainly focuses on determining the change in optical and electrochemical parameters i.e., current, potential, voltage, colour, phase change, etc., which comes under the class of opto-electrochemical biosensors. Furthermore, we have explored the labelled and label-free aptasensors for the detection of viral infections. The labelled and label-free aptasensors are classified according to the different signal readout system, for instance electrochemical and optical signals. Moreover, the globally developed aptasensors for the virus monitoring have been comprehensively discussed and represented in various figures. Significant attention is given to the fabrication process and how the aptamers are incorporated in the design of aptasensors for various targets. Various fabricated aptasensors, especially in the last 3 years, have been explained in tabulated form, describing their readout system and analytical details, such as dynamic range, limit of detection (LOD), aptamer sequence, labelling molecule, etc. Finally, we summarise and present an insight towards the future of aptasensors, speculating on how they will become more convenient, cost-effective, rapid test devices.

## 2. Label and Label-Free Aptasensors

### 2.1. Aptamers and Biosensors

Aptamers are single-stranded oligonucleotide sequences (DNA or RNA) that may fold into a stable three-dimensional (3D) structure and interact with a target molecule, both sterically, and through electrostatic interactions. The electrostatic complementarity occurs because of an interaction between the positive and negative charges present on the surfaces of the aptamer and the target. Apart from the electrostatic interaction, other intermolecular interactions such as van der Waals forces, hydrogen bonds and π-π stacking make the aptamer–target complex stable [25]. They feature distinct characteristics such as compact size, low cost, high specificity, facile chemical modification, and most importantly extraordinary flexibility. These are capable of binding specific targets with high affinity, specificity, and sensitivity [26,27]. The target molecule can be as simple as a metal ion, and can be as complex as large protein molecules such as cardiac troponin, or even cells [28,29,30,31,32]. Aptamers are designed by an easier, cheaper in vitro method called SELEX (systematic evolution of ligands by exponential enrichment) [33,34]. This simple but elegant technique was discovered by a group of scientists—Larry Gold and Jack Szostak—and published for the first time in 1990 [35,36]. Because of the in vitro selection method of aptamers, this allows us to engineer the affinity molecules which are non-immunogenic or toxic to the cells. The chemical synthesis method of aptamers is easy and cost-effective, and warranties a high-level consistency from batch-to-batch [37]. Aptamers show numerous advantages over antibodies, including a low cost of development, high stability, sensitivity, and an easier modification process. In addition, they are not immunogenic [38,39,40,41]. Moreover, their insensitivity towards any change in pH, temperature, and ionic strength make them a preferred choice over antibodies. In the year 1996, fluorescently labelled aptamer was first reported to be utilized as a bio-detection agent in biosensors [42,43,44,45]. In the last decade, the implementation of aptamers has increased exponentially in the design of biosensors. This exponential increase is concluded from a scientific survey performed on SCOPUS for the number of research articles published with the keyword “aptasensor”, especially in last decade (Figure 1B).

**Figure 3:**
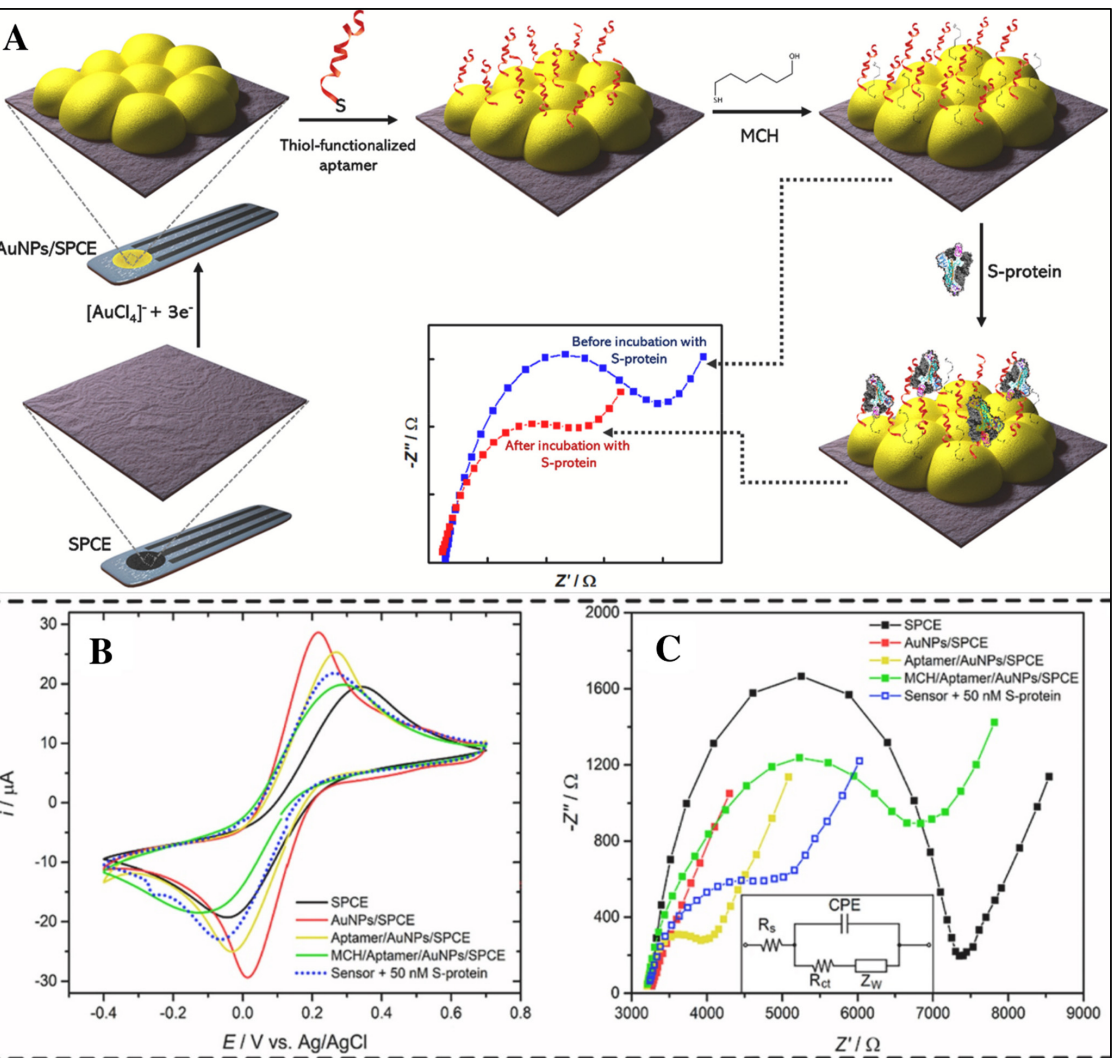
(**A**) Step-by-step fabrication of aptasensor for the detection of SARS-CoV-2 by targeting the receptor-binding domain. (**B**) Representation of CV. (**C**) EIS results for every step of the aptasensor fabrication and detection of SARS-CoV-2 (reprinted with permission from [67]. Copyright 2021 Elsevier).

Biosensors are analytical devices that provide detection of target analytes, both qualitatively and quantitatively [38,39,40]. Biosensors show excellent potential to fulfil the ASSURED criteria (i.e., affordable, sensitive, specific, user-friendly, rapid and robust, equipment-free and deliverable to users) for developing point-of-care devices [46]. There are three essential components of any type of biosensor, i.e., BRE, transducer, and an amplifier and processor. The selection of ideal substrate is also one of the important and challenging tasks the in fabrication of biosensors [22]. On the basis that transduction system biosensors can be categorized in different classes such as electrochemical, optical, mechanical, etc. [47,48,49,50], in this study, a detailed focus is given on the optical and electrochemical aspect of the transduction system. Electrochemical biosensors measure the signal based on any minute difference in potential, conductance, current, or field effect that occurs because of the binding between the BRE and the analyte [51,52]. These systems are preferred over others because of their selectivity, sensitivity, and operational details [53,54,55,56]. The three electrodes typically used in an electrochemical system include; the working electrode (e.g., glassy carbon electrode functions as the transducing element); the auxiliary/counter electrode (e.g., Pt electrode, acts to complete the circuit); and the reference electrode (e.g., Ag/AgCl electrode, important for creating a steady potential) [24,57,58,59]. The important task in this system is selecting the suitable working electrode according to the need for modification and surface nano-engineering to target a specific analyte. The basic principle behind the working of such biosensors is the electron or ion transfer kinetics from the reaction point to the surface of electrode [60,61,62]. In both labelled and label-free electrochemical biosensors, any change (positive or negative) in electron transport is monitored. Numerous techniques have been developed to study the change in various parameters (potential, current, charge, time, conductivity, impedance), for instance, Cyclic Voltammetry (CV), chronoamperometry, differential pulse voltammetry, square wave voltammetry, etc. Another electrochemical technique, i.e., electrochemical impedance spectroscopy (EIS), is used to study the electrochemical kinetics of sensing surfaces through inducing the electrochemical kinetics on working electrodes [63,64,65,66]. All these techniques aid in detecting any changes occurring on the surface of electrodes, such as whether the BRE (such as the aptamer) has bound or not. For instance, an electrochemical aptasensor for the detection of SARS-CoV-2 has recently been designed by Abrego-Martinez et al., in 2021. In this sensor, screen-printed carbon electrode (SPCE) was used and modified with AuNPs, after which an aptamer was immobilised on the electrode surface illustrated in Figure 3A. The change in electrochemical parameters with every step of modification on SPCE and the fabrication of the aptasensor was monitored by CV and EIS, as shown in Figure 3B,C, respectively. All these recordings were performed in phosphate buffer saline solution containing 5 mM [Fe(CN)_6_]^3−/4−^ [67].

**Figure 4:**
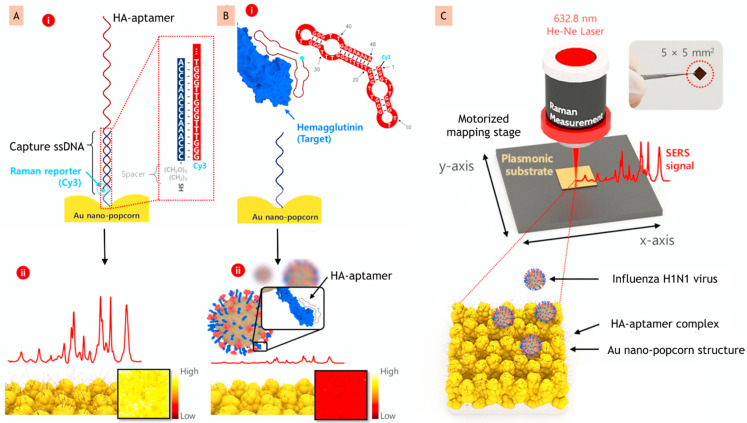
(**A**) (i) Aptamer probe labelled with Cy-3 on the surface of nano-popcorn; (ii) strong Raman signal created by labelled structure. (**B**) (i) Representation of conformational change that occurred due to recognition of A/H1N1 virus; (ii) effect of recognition on Raman signal. (**C**) Schematic representation of the SERS-based aptasensor by utilizing a 3D nano-popcorn for the detection of A/H1N1 virus quantitatively (reprinted with permission from [71]. Copyright 2020 Elsevier).

Optical biosensors allow us to analyse the polarity, phase or frequency change in the optical field of a BRE, due to interaction with the target. Optical biosensors can further be categorized into luminescence, absorption, or fluorescence-based, on the basis of the transduction mechanism [68]. Optical biosensors based on change in refractive indexes are one of the most preferred, because of their great performance, low cost, and user-friendly nature. This type of sensors helps in the miniaturization of devices due to the absence of complex reactions, and in developing multiplexing devices [69,70]. In this class, the direct detection or naked-eye-based optical system (labelled and label-free) are common, due to their simple and fast detection procedure. For instance, Chen et al., 2021, have developed an aptasensor based on surface-enhanced Raman scattering (SERS) for the detection of influenza virus A (H1N1) virus by immobilizing a labelling probe, i.e., Cy3 with an aptamer on the nanostructure surface. A strong Raman signal was generated when the Cy3 label attached to the aptamer and a further interaction of the label with the target (Hemagglutinin) shows the reduction in Raman signal, as illustrated in Figure 4A,B, respectively [71].

### 2.2. Labelled Opto-Electrochemical Aptasensors for Virus Detection

Aptasensors are classified into two types, namely, labelled and label-free, depending upon whether the labelling molecule is used for signal enhancement/generation or not. A label is a foreign molecule which is bonded through chemical or physical means to monitor the target analyte. Numerous kinds of molecules are used as labels, for example, fluorophores, enzymes, dyes, etc. In the earlier days of biosensors development, various kinds of optical or radioactive molecules were used as labels inspired from conventional electrophoresis ELISA techniques [72,73,74]. Label-based sensing systems utilize various types of transducing systems for signal detection, such as colorimetric, electrochemical, electro-chemiluminescence, SERS, etc. Labels are employed in electrochemical biosensors to boost the signal produced per occurrence. Electrochemical labelling often involves chemical methods that covalently bind labels, but some probe labelling merely requires momentary (physical-binding) attachments [75,76,77]. In one of the fascinating studies, Karash et al. designed an impedance-based aptasensor utilizing a specific avian influenza virus (AIV) H5N1 aptamer and a gold interdigitated microelectrode. In this, the biotin-tagged H5N1 aptamer was coupled to incorporate streptavidin on the microelectrode surface. The virus was captured by the attached aptamer after polyethylene glycol was used as a blocking agent on the microelectrode surface. Furthermore, an amplifier was prepared based on nanoparticles and applied to improve the impedance signal by establishing a pattern-like AuNP/H5N1-aptamer/thiocyanuric acid system. The impedance aptasensor showed a detection limit of 0.25 HAU (hemagglutinations unit) for pure virus, and for H5N1 virus-spiked tracheal chicken swab samples the limit was 1 HAU. The system was able to explicitly detect the subtype H5N1 using specialized aptamers that bound to the virus, but not the nonspecific virus. This aptasensor was proven to boost impedance signal change by at least 48 times, laying the groundwork for the creation of a portable and cost-effective technique [78]. Similarly, in another work, Xu et al. developed a fluorescent-based hydrogel aptasensor for the fast sensing of H5N1. An aptamer was selected specifically for H5N1, and two partially complementary single-stranded DNAs (ssDNA1 and ssDNA2) were created for the attachment to aptamer’s two ends. Furthermore, for hydrogel production via polymerization, both aptamer and ssDNA1 were functionalized with acrydite at the 5′-terminus. Quantum dots (QDs) were tagged at the 5′-terminal of ssDNA2 as fluorescence reporters, and at the 3′-terminal of the aptamer, quenchers for QDs were coupled. The crosslinker for the QD-aptamer hydrogel was generated by hybridization between the aptamer and ssDNAs. When the target binds, the crosslinking between the aptamer and target get segregated, because of the binding reaction between them, causing the hydrogel to suddenly inflate and the liberation of the aptamer-quencher and ssDNA2-QDs. The quartz crystal microbalance method was used as a readout system to reveal the aptamer’s responsiveness to target binding. From sampling to results, it took 30 min to complete the detection process. The detection range was found to be 2^−1.2^–26 HAU 20 µL^−1,^ with an LOD of 0.4 HAU. The suggested method is a quick, simple, specific and label-free assay, that can be expanded further as a generic and practical detection platform, with benefits such as: (1) when specific aptamers are available, QDs with distinct emission wavelengths can be used to detect several targets at the same time; (2) the idea of developing a portable apparatus could be realized through the dropping of hydrogel in a solid substrate for detection; (3) the properties which are affected by the size of the hydrogel-based aptasensor provide a viable technique to develop a recognition pattern of aptamer-hydrogel with an optimal pore size and crosslinking density for certain analytes [79].

Apart from the implication of labelled aptasensors in H5N1 detection, they are also exploited for the detection of other viruses. For instance, in a recent study, Chen et al. developed a SERS imaging-based aptasensor for the sensitive and repeatable spotting of H1N1 by targeting the Hemagglutinin protein [71]. They used the difference in surface energy between a spacer (made of perfluoro decanethiol) and a Au layer to fabricate a plasmonic substrate of 3D nano-popcorn (Figure 4C). This differential energy caused Au nanoparticles to self-assemble, resulting in numerous hotspots on the substrate. To obtain a robust Raman signal, the aptamer probe was labelled with Cy3 and hybridized with thiolated capture DNAs anchored on the 3D nano-popcorn surface. The incident field was greatly amplified by localized surface plasmon effects in the hotspot areas. The LOD and assay duration of the developed A/H1N1 virus aptasensor were estimated to be 97 PFU (Plaque Forming Unit) mL^−1^ and 20 min, respectively. PFU is mainly used in virology as a measure to describe the number of virus particles capable of forming plaques per unit volume. The suggested SERS-based image aptasensor platform solves the drawbacks of traditional techniques, i.e., time-consuming, labor-intensive RT-PCR (reverse transcriptase-polymerase chain reaction), minimal sensitivity and quantitative analytical limits of lateral flow assay kits. Mok et al., 2021, designed a one-shot labelled aptasensor for the detection of the Dengue virus non-structural protein (NS1). In this study, a G-quadruplex-forming Dengue virus-derived NS1-binding aptamer (DBA) was developed and further labelled with a fluorescent dye 6-carboxy fluorescein (FAM) at the 5′ end. Optical sensing is achieved by the detection of the structural destruction of DBA, which is induced by the NS1 protein. The 5′FAM-DBA quantitatively detects the fluorescence quenching brought about by Guanines upon NS1 binding. The constructed one shot, simple, rapid aptasensor achieved an LOD of 8.13 nM and a wide dynamic range of 2.81 nM to 360 nM [80]. Because of the enormous impact of aptamers, different groups of researchers are working on exploring this field, and aptasensors are being developed worldwide for different targets. Therefore, apart from the above explained examples, various other developed labelled aptasensors have been mentioned in Table 1, describing the target, labelling molecule, aptamer sequence, and analytical details.

### 2.3. Label-Free Opto-Electrochemical Aptasensors for Virus Detection

The labelling process not only affects the aptamer’s target-binding affinity, but also adds operational complexity and cost. As a result, for sensing applications, a label-free method is more preferred as it helps in the detection of target molecules in their natural form, because they are not altered through labelling procedure [92,93,94]. The BRE is not labelled with redox chemicals in label-free techniques. Instead, the aptamers are functionalized on the electrode surface, and electrochemical methods (for instance CV and EIS) are used to identify the target–aptamer interaction. Electroactive compounds for instance ferricyanide and ruthenium complexes ([Fe(CN)_6_]^3−/4−^ and Ru(NH3)_6_]^3+^) that may attach electrostatically or interact diffusively (attraction/repulsion) with the aptamers are used in these electrochemical approaches. As these systems are more reliable and effective, researchers are exploring label-free aptasensors worldwide for the detection of different categories of biochemical molecules, bacteria, viruses, and mammalian cells. In this section, we focus on recent studies, especially in last 3 years, for the detection of various classes of viruses that are creating a major impact on human health and the economy. One such example is the aptamer-dependent detection of dengue virus belonging to the *Flavi* virus family causing dengue fever. This is one of the endemic diseases in tropical and subtropical regions, affecting around 40% of the world population [95]. The existing diagnostic methods of dengue fever are considered as expensive, time-consuming, and too complex to be used in remote areas, especially in places where infrastructure is not well established. Although many POC devices have been developed so far, they show some limitations relating to time consumption, and even specificity and selectivity [96,97]. To overcome this situation, for the first time in the year 2020, Rashid et al. have fabricated an electrochemical aptasensor for the detection of Dengue fever by targeting its NS1 protein [98]. NS1 is a non-structural protein which is considered as a specific biomarker for dengue fever [99,100]. The device was fabricated by targeting the specific interaction between the NS1-aptamer and the polymer-induced AuNPs. Cationic polymers, i.e., polyethyleneimine, aptamer and NS1, were deposited on the AuNPs, and this aggregation formed a duplex structure. The structural transition between the aptamer/polymer duplex and the aptamer/target complex produced the electrochemical signal. The developed aptasensor showed the improved dynamic range between 3–160 ng mL^−1^ with a LOD value of ~0.3 ng mL^−1^. The cost-effectiveness and inherent flexibility of this method could be utilized to pave a way towards the effective diagnosis of Dengue virus and other infectious diseases. Recently, in the year 2021, Junior et al. have designed a more sensitive electrochemical aptasensor for targeting the same. DNA aptamers and 6-mercapto-1-hexanol were immobilised on the surface of gold electrodes to form a self-assembled monolayer. The ratio of aptamer and 6-mercapto-1-hexanol on the surface was optimised to obtain the enhanced signal and the modified electrode surface was characterised by EIS and atomic force microscopy. The non-specific interaction on the surface was blocked by using bovine serum albumin that helps in stabilising the NS1 solution. The sensor showed different LODs of 0.05, 0.022 and 0.025 ng/mL, based on the serotype samples [101].

**Figure 5:**
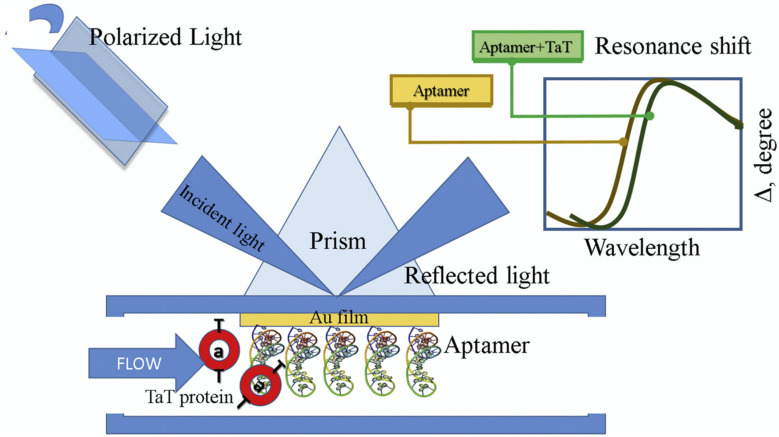
Pictorial representation of RNA aptasensor for the detection of HIV-Type 1 based on spectrophometric ellipsometry by targeting Tat protein (reprinted with permission from [105]. Copyright 2019 Elsevier).

Infectious diseases are becoming the major driver of high morbidity and mortality rates worldwide. One of the major contributors to this scenario are viral infections. Human immunodeficiency virus (HIV) is amongst the top 5 contributors to infectious diseases globally [102,103,104]. Thus, there is an urgent requirement to find capable and impactful strategies that help towards the better treatment of such infectious diseases. The first step towards achieving this is the simple, cost-effective and fast detection method of such infectious diseases. In the year 2020, Caglayan et al. tried to monitor HIV Type 1 by developing an optical aptasensor. The aptasensor targeted the Tat (trans-activator of transcription) protein that consisted of 101 amino acids and played a key role in controlling the first stage of the replication cycle of HIV-1. The detection was based on the interaction between HIV-Tat protein and the anti-Tat aptamer that caused changes in spectroscopic ellipsometry and in the surface plasmon resonance-enhanced total internal reflection ellipsometry (Figure 5). Ellipsometry is a well-known phenomenon for the characterisation of ultra-thin-film, in which the change in polarisation state shows surface deposition in terms of the ellipsometric angles, psi (Ψ) and delta (Δ). The developed device shows a wider dynamic range of 1.0–500 nM with an LOD of 1 pM [105]. In 2021, another study was conducted for the detection of HIV, targeting a different binding site, i.e., p24-HIV protein-using aptasensor technology [106]. The aptasensor was designed by using a screen-printed electrode which was modified through the dispersion of graphene quantum dots (GQD) by electrodeposition. This modification helps in promoting the reduction of the oxygenated groups present on the surface of material, decreases solubility and enhances depositions of GQD. Furthermore, the aptamer was immobilized onto the electrode, forming a covalent interaction between the carboxylic group of material and the amino group of aptamers. The p24-HIV protein was measured by the electrochemical method and the fabricated aptasensor showed a dynamic range of 0.93 ng mL^−1^ to 93 mg mL^−1^ and an LOD of 51.7 pg mL^−1^.

Similarly, hepatitis C virus (HCV) infection is also a health burden worldwide, with around 170 million chronically infected people. HCV mainly infects the hepatocytes and enter the body through hepatic sinusoids causing progressive liver disease. Therefore, utilizing the aptamer-based technology in the year 2017, an electrochemical aptasensor was designed for the ultra-sensitive detection of HCV by Ghanbari et al. The aptasensor was designed by modifying glassy carbon electrodes through the immobilisation of GQDs on its surface. GQDs proved to be a more suitable substrate for aptamers, due to π-π stacking interactions and the hydrophobic plane that helps in improving the aptamer absorption on the surface of electrode. The developed device directly targets the core antigen of the HCV, and the EIS method was used to confirm the monitoring process. The sensor shows two dynamic ranges, i.e., 10–70 pg mL^−1^ for a lower concentration, and 70–400 pg mL^−1^ for a higher concentration of the HCV core antigen, with a detection limit of 3.3 pg mL^−1^. The accuracy and efficiency of the aptasensor has also been checked in human serum samples, showing promising results [107]. In a recent study, Rahmati et al., 2021, designed an advanced and more sensitive electrochemical-based aptasensor for the detection of HCV [108]. In this detection system, the porous 3D-NiCo_2_O_4_ nanowire was bombarded in an N-doped carbon thin layer, showing morphology like a sea urchin, as confirmed by field emission scanning—electron microscopy (FE-SEM). The generated N-C@NiCo_2_O_4_ nanowire combines the electrochemical benefits of Co and Ni species which show significantly specific active sites, greater capacitance, and better conductivity. More importantly, the layer of carbon helps in improving nanowires’ stability, porosity and conductivity. The 3D N-C@NiCo_2_O_4_ nanowires were used to augment the load of the HCV core antigen aptamer on the surface of the electrode, resulting in a substantial improvement in sensitivity. The developed aptasensor shows a dynamic range between 0.5 fg mL^−1^ to 0.12 pg mL^−1^ with an LOD of 0.16 fg mL^−1^.

Another virus majorly responsible for substantial economic loss globally is the Norovirus, which causes acute viral gastroenteritis. Noroviruses are infectious, and possesses high thermal stability and an ineffectiveness towards more common disinfectants and sanitizers. It becomes necessary to develop a fast, selective, sensitive and cost-effective bioanalytical sensing prototype for Norovirus detection. Recently, in 2021, Jiang et al. developed an electrochemical 3D aptasensor for the analysis of norovirus [109]. The SPCE was used, and a working electrode was developed by covering the head of the ball with the carbon ink of a pin, which made a 3D hybrid electrochemical aptasensor. This design of movable spherical working electrodes assisted in increasing specificity. The working electrode was further patterned with phosphorene-gold nanocomposites, and the nano composite was fabricated by the process of in situ reduction of phosphorene nanosheets on chloroauric acid. The developed device shows a broad dynamic range of 1 ng mL^−1^–10 μg mL^–1^ and an LOD of 0.28 ng mL^−1^. Table 2 describes the target transduction system, aptamer sequence, dynamic range, LOD, etc.

### 2.4. Aptasensors for COVID-19 Detection

In late 2019, the outbreak of the novel severe acute respiratory syndrome coronavirus 2 (SARS-CoV-2) led to the COVID-19 pandemic that has drastically impacted the human population, leaving the world clueless, and in an unimaginable situation. PCR, which detects the viral RNA, and serological screening by detecting antibodies generated in response to the infection, are the two primary screening procedures for confirming SARS-CoV-2 [125,126,127,128]. However, new methods are urgently needed due to the detection complexity, expense, and relatively longer analysis period of the present approaches. Therefore, aptasensor technology has been explored to prepare a rapid, selective, and sensitive method for the identification of SARS-CoV-2. In one of the studies, a D-shaped plastic optical fibre (POF)-based aptasensor has been designed to detect the spike protein of SARS-CoV-2. A specific aptamer sequence was immobilized on polyethylene glycol, which was further deposited on AuNPs, attached on POFs. The author utilizes the high sensitivity of surface plasmon resonance for monitoring the protein binding on the POF probe. The designing of the aptasensor for SARS-CoV-2 glycoprotein was performed by targeting its receptor-binding domain. The aptasensor had offered a considerably lower LOD of about 37 nM. The specificity of the designed aptasensor was also checked by confirming the detection within the various similar interfering molecules, such as the MERS spike protein. This type of sensing design encourages further developments, with the aim of producing a small size, portable laboratory diagnostic tool [90]. In the ongoing scenario, another aptasensor has been reported for the detection of SARS-CoV-2, targeting the receptor-binding domain in the spike protein (S-protein). The incubation time of the aptamer, target, and potential pulse time for AuNPs deposition was initially examined by using the glassy carbon electrode. The aptamer was decorated on AuNPs, and the detection of the S-protein in the complex of aptamer–target was performed using the photo-induced force microscopy mapped from 770 to 1910 cm^−1^. EIS was used for the final detection of the S-protein of SARS-CoV-2 after the incubation time of 40 min. The device shows the acceptable analytical parameters, including an LOD of 1.30 pM (66 pg/mL) [67]. Similarly, the Tabrizi group have developed an electrochemical aptasensor for SARS-CoV-2 by targeting its receptor-binding domain [119]. Quantum dots were used in sensor fabrication by modification with graphitic carbon nitride (gC3N4) and cadmium sulphide (CdS). The CdS QDs-gC3N4 nanocomposite was dissolved in a chitosan-containing solution to form Chitosan/CdS-gC3N4 nanocomposite. The developed aptasensor shows a measurable range of 0.5–32.0 nM, and an LOD of 0.12 nM.

**Figure 6:**
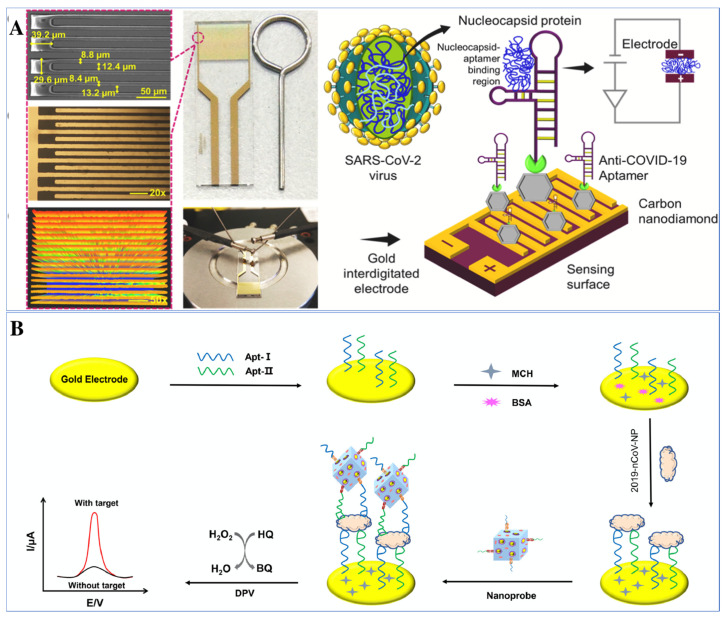
Illustration of electrochemical aptasensors for the detection of SARS-CoV-2. (**A**) Schematic of nucleocapsid protein detection of SARS-CoV-2 through immobilization of anti-NCP aptamer on diamond-enhanced gold interdigitated electrode (reprinted with permission from [118]. Copyright 2021 Elsevier) (**B**) Representation of dual-aptamer-based biosensor for nucleocapsid protein detection of SARS-CoV-2 by utilizing metal organic framework decorated with Au@Pt nanoparticle (reprinted with permission from [88]. Copyright 2021 Elsevier).

Ramanathan et al. developed a portable POC aptasensing device in 2021, for the impedimentary-based identification of SARS-CoV-2 by targeting its nucleocapsid protein (NCP). The system was fabricated by utilizing a 10 μm gap-sized gold interdigitated electrode (AuIDE) and the electrode surface was improvised with a silane group. Furthermore, ~20 nm of diamond was deposited on the modified electrode surface which helped in enhancing the detection of NCP (Figure 6A). The characterization of the diamond-enhanced AuIDE was performed by using XRD, XPS and FTIR analysis. EIS was implemented for the evaluation of SARS-CoV-2 NCP in a spiked human serum sample. The fabricated aptasensor shows good selectivity, linear dynamic ranges from 1 fM to 100 pM, and a lower LOD of 0.389 fM. The aptasensor was also checked for its stability and reusability and showed ~30–33% of activity loss after 11 days of analysis. Not only that, the interaction between NCP aptamer and protein was also confirmed through ELISA [118].

In addition to these, labelled aptasensing mechanisms have also been utilized for the detection of SARS-CoV-2. Tian et al. 2021 have developed a labelled aptasensor for the detection of SARS-CoV-2 by targeting its NCP. A dual aptamer based electrochemical prototype was constructed by using metal organic frameworks MIL-53 (Al) deposited on Au@Pt nanoparticles. Firstly, the two aptamers, i.e., N48 and N61, have been immobilized on the gold electrode surface to target the biomarker of nCoV, i.e., NCP. The Au@Pt/MIL-53 composites decorated with horseradish peroxidase and hemin/G-quadruplex DNAzyme were used as a signal nano probe (Figure 6B). The nanoprobe was used to enhance the signal of the aptasensor in the presence of hydrogen peroxide through the co-catalysed oxidation of hydroquinone. The detection system, i.e., the aptamer-protein-nanoprobe, demonstrated a broad linear range from 0.025 to 50 ng mL^−1^ with an LOD of 8.33 pg mL^−1^ [88].

After a detailed study of recently published papers, with particular focus on the last 3 years, of label and label-free aptasensors focusing on optical and electrochemical readout systems, we are able to provide a comparison between these two systems. The study was mainly carried out by specifying the target, i.e., viruses, as viral infection has become one of the major infectious diseases throughout the world. Hence, in Table 3, the advantages and disadvantages of these two methods (optical and electrochemical) are tabulated to provide a clearer idea towards the aptasensors.

## 3. Conclusions and Future Perspective

The growth of the biosensors industry is vast and tremendous, as a large number of devices are either already commercially available, or being developed in academic laboratories. The advancement of different techniques, and the emergence of rapid prototyping methods have helped towards the tremendous growth of biosensors in the last 60 years, but still this field has a long way to go. During the sensing process, there is a major role played by the BRE, this could be enzymes, antibodies, nucleic acids, aptamers, etc. Initially, antibodies were predominantly used as BRE, but they were accompanied by various other issues, i.e., higher production cost and time, immunogenicity, etc. Thus, industries and scientists needed a more suitable, specific and cost-effective BRE, that leads to the emergence of aptamers. Aptamers prove to be an excellent BRE in every aspect, ranging from greater specificity to cheap production. Viruses are one of the most infectious, deadly disease-causing agents which impact the human population worldwide. The implication of aptamers in developed sensors for the detection of different kinds of viruses has been explained and discussed in detail in this review. Different globally developed aptasensor utilizing various nanomaterials and targeting different classes of viruses are illustrated and discussed in tabulated form, to provide a broader idea to the readers. The above explained examples highlight many promises to develop rapid, cheap, and specific aptasensing prototypes. Even after such enormous growth and development, commercial availability of these devices is still lacking. During the time of COVID-19 pandemic, laboratorial methods are used in most places, initially. Even after the development of numerous aptasensors and the commercialization of a few kits, traditional methods are still in use, especially in developing and under-developed countries. Therefore, to encounter the various issues related with aptasensors and to maximise their impact, we need to overcome several issues: first, minimising the cost of aptasensors to improve their use in developing countries; second, the selection and designing procedure to make specific aptamers need to be simplified. Finally, researchers need to focus on new technologies that can help in creating more advanced, user-friendly rapid detection devices.

## Figures and Tables

**Table 1:** Labelled opto-electrochemical aptasensor for virus detection (NR—not reported).

Sl.No	Target	Target Genetic Material (RNA/DNA)	Labelling Molecule	Aptamer Sequence	Binding Description	Detection Range	LOD	Detection Method	References
1	H1N1	RNA	Cy3 (Cyanine dye 3)	Probe: 5′-Cy3/GGGTTTGGGTTGGGTTGGGTTTTTGGGTTTGGGTTGGGTTGGGAAAAA-3′ Capture: 5′-ACCCAACCCAAACCC-(CH_2_O)_3_(CH_2_)_3_-SH-3′	Target induces aptamer to form DNA duplex	10–10,000 PFU mL^−1^	97 PFU mL^−1^	SERS	[71]
2	Influenza virus	RNA	Cy3	Primary aptamer: 5′-HS-(CH_2_)_6_-TTGGGGTTATTTTGGGAGGGCGGGGGTT-3′ Secondary aptamer: 5′-Cy3-TTG GGGTTATTTTGGGAGGGCGGGGGTT-3′	Aptamer binds to the surface of target	2.5 × 10^−4^–1.3 HAU mL^−1^	1 × 10^−4^ HAU mL^−1^	SERS	[81]
3	Influenza virus	RNA	BODIPY FL	5′-HS-(CH_2_)_6_-TTGGGGTTATTTTGGGAGGGCGGGGGTT-3′	Target induces aptamer to form DNA duplexes	2 × 10^5^–2 × 10^6^ VP mL^−1^	2 × 10^5^ Viral particles mL^−1^	SERS	[82]
4	HIV	RNA	Europium sulfide nanocrystals (EsNCs)	5′-NH_2_-GGGGGGCCAAGGCCCAGCCCTCACACA-3′	Target induces ssDNA aptamer to form DNA duplex	3.0 fM–0.3 nM	0.3 fM	Electrochemiluminescence	[83]
5	HBV	DNA	Methylene Blue	5′ -SH-(CH_2_)_6_-GGGAATTCGAGCTCGGTACCGGCACAAGCATATGGACTCCTCTGAACCTACGATGTAGTACCTGCAGGCATGCAAGCTTGG-3	Target induces ssDNA aptamer to form DNA duplex	0.125–2.0 fg mL^−1^	0.0014 fg mL^−1^	Electrochemical	[84]
6	HBV	DNA	ALP-labeled Streptavidin	S1: 5′-CACAGCGAACAGCGGCGGACATAATAGTGCTTACTACGAC-3′ S2: 5′-CGAGCTCGAATTCCCGATCTCTAG-SH-3′ S3: 5′-Biotin-TCGCAGTGT-SH-3′	Aptamer binds to target surface	1–225 ng mL^−1^	0.05 ng mL^−1^	Chemiluminescence	[85]
7	Flavivirus	RNA	6-carboxyfluorescein (FAM)	5′-FAM-AGCGGATCCGATGGGTGGGGGGGTGGGTAGGATCCGCG-3′	Target induces aptamer structure (G-Quadruplex) destruction	2.81 nM–360 nM	8.13 nM in serum.	Fluorometric	[80]
8	Norovirus	RNA	6-carboxyfluorescein	5′-AGTATACGTATTACCTGCAGCCCATGTTTTGTAGGTGTAATAGGTCATGTTAGGGTTTCTGCGATATCTCGGAGATCTTGC-3′	Binding of aptamer to the target surface	13 ng mL^−1^–13 μg mL^−1^	4.4 ng mL^−1^ (MWCNT) 3.3 ng mL^−1^ (GO)	Fluorometric	[86]
9	MERS-CoV-2	RNA	Methylene blue	S-19 aptamer: 5′-TGACACCGTACCTGCTCTGCACTTCCTTCACCAGAAACCTGCACATCTTCGCCGCGTGAAGCACGCCAAGGGACTAT-3′	Aptamer targets the S protein	1 pg mL^−1^–1 ng mL^−1^ 1 pg mL^−1^–1 ng mL^−1^	0.525 pg mL^−1^ 0. 645 pg mL^−1^	Electrochemical SERS	[87]
10	SARS-CoV-2	RNA	HRP and hemin/G quadruplex DNAzyme	NR	Target induces aptamer to form G-quadruplexes	0.025–50 ng mL^−1^	8.33 pg mL^−1^	Electrochemical	[88]
11	SARS-CoV-2	RNA	Cy3 Raman reporter	Probe: 5′-Cy3/TTTTTTTTTTTTTTTCAGCACCGACCTTGTGCTTTGGGAGTGCTGGTCCAAGGGCGTTAATGGACA-3′ Capture: 5′-AAAAAAAAAAAAAAA- (CH_2_O)_3_(CH_2_)_3_-SH-3′	Aptamer targets the receptor-binding site	0–1000 PFU mL^−1^	<10 PFU mL^−1^	SERS	[89]
12	SARS-CoV-2	RNA	Cy3-Streptavidin (Cy3-SA)	NR	Aptamer targets receptor-binding domain	NR	37 nM	Fluorometric	[90]
13	SARS-CoV-2	RNA	Nickle beads (Ni-beads)	5′-CAGCACCGACCTTGTGCTTTGGGAGTGCTGGTCCAAGGGCGTTAATGGACA-3′ 5′-ATCCAGAGTGACGCAGCATTTCATCGGGTCCAAAAGGGGCTGCTCGGGATTGCGGATATGGACACGT-3′	Aptamer targets receptor-binding domain	NR NR	5.8 nM 19.9 nM	Fluorometric Fluorometric	[91]

**Table 2:** Label-free opto-electrochemical aptasensor for virus detection.

Sl.No	Target	Target Genetic Material (DNA/RNA)	Aptamer Sequence	Binding Description	Detection Range	LOD	Detection Method	References
1	p24-HIV	RNA	NR	Aptamer binds to capsid protein of target	0.93 ng mL^−1^–93 mg mL^−1^	51.7 pg mL^−1^	Electrochemical	[106]
2	Flavivirus	RNA	5′-HS(CH_2_)_6_-TTTTT-ACTAGGTTGCAGGGGACTGCTCGGGATTGCGGAT CAACCTAGTTGCTTCTCTCGTATGAT-3′	Aptamer binds to the surface of target	0.01–100 ng mL^−1^	0.022 ng mL^−1^	Electrochemical	[101]
3	HCV	RNA	5′-NH_2_-ACTATACACAAAAATAACACGACCGACGAAAAAACACAACC-3′	Aptamer binds to target surface	0.5 fg mL^−1^–0.12 pg mL^−1^	0.16 fg mL^−1^	Impedimetric	[108]
4	Inactivated H1N1	RNA	NR	Multivalent binding of aptamer to target	NR	0.9 pg μL^−1^	Electrochemical	[110]
5	H1N1	RNA	5′-TACTGCACACGACACCGACTGTCACCATCACCTCGGCGCA-3′	Aptamer binds to surface of target	10^1^ PFU mL^−1^–10^4^ PFU mL^−1^	3.7 PFU mL^−1^	Electrochemical	[111]
6	Norovirus	RNA	5′-AGTATACCGTATTACCTGCAGCCATGTTTTGTAGGTGTAATAGGTCATGTTAGGGTTTCTGCGATATCTCGGAGATCTTGC-3′	Aptamer targets capsid protein of target	100 pM–3.5 nM	100 pM	Electrochemical	[112]
7	Norovirus	RNA	5′-SH-(CH_2_)_6_-GGGAATTCGAGCTCGGTACCG GCACAAGCATATGGACTCCTCTGAACCTACG ATGTAGTACCTGCAGGCATGCAAGCTTGG-3′	Aptamer binds to surface of target	0.25 fg mL^−1^–1.5 fg mL fg mL^−1^	0.018 fg mL^−1^ (CV), 0.0016 fg mL^−1^ (SWV) and 0.001 fg mL^−1^ (EIS)	Electrochemical	[113]
8	Murine Norovirus	RNA	5′-GCTAGCGAATTCCGTACGAAGGGCGAATTCCACATTGGGCTGCAGCCCGGGG GATCC-3′	Target induces aptamer to desorp from the surface	200–10,000 viruses mL^−1^ 1320–19,800 viruses mL^−1^ 3300–33,000 viruses mL^−1^	30 virusesmL^−1^ 50 virusesmL^−1^ 80 viruses mL^−1^	Colorimetric	[114]
9	Zika	RNA	5′-ThioMC6-D-AGCC ATGACCGACACCACACCGT-3′	Aptamer binds to the surface of target	1.0 × 10^−12^–1.0 × 10^−6^ mol L^−1^	0.82 pmol L^−1^	Electrochemical	[115]
10	HCV	RNA	5′-CTATACACAAAAATAACACGACCGACGAAAAAACACAACC-3′	Aptamer targets the core antigen	5 fg mL^−1^–1.0 pg mL^−1^	1.67 fg mL^−1^	Electrochemical	[116]
11	Papillomavirus	RNA	5′-GGGAACAAAAGCUGCACAGGUUACCCCCGCUUGGGUCUCC-3′	Aptamer binds to surface of the target	9.6–201.6 ng mL^−1^	9.6 ng mL^−1^	Colorimetric	[117]
12	SARS-CoV-2	RNA	NR	Aptamer binds to the nucleocapsid binding region	1 fM–100 pM	0.389 fM	Electrochemical	[118]
13	SARS-CoV-2	RNA	5′-NH_2_-(CH_2_)_6_-CAGCACCGACCTTGTGCTTTGGGAGTGCTGGTCCAAGGGCGTTAATGGACA-3′	Aptamer binds to the RBD of the target	0.5–32.0 nM	0.12 nM	Electrochemical	[119]
14	AIV H5N1	RNA	NR	Aptamer binds to the surface of the target	0.128–1.28 HAU	0.128 HAU	SPR	[120]
15	SARS-CoV-2	RNA	5′-MeBlN/CAGCACCGACCTTGTGCTTTGGGAGTGCTGGTCCAAGGGCGTTAATGGACA/3ThioMC-3′	Aptamer targets RBD	NR	1 ag mL^−1^	Electrochemical	[121]
16	SARS-CoV-2	RNA	5′-dithiol-CAGCACCGACCTTGTGCTTTGGGAGTGCTGGTCCAAGGGCGTTAATGGACA-3′	Target induces receptor-binding domain	NR	0.09 (for 99% of aptamer)	SERS	[122]
17	SARS-CoV-2	RNA	S1 Aptamer: 5′-Biotin-CAGCACCGACCTTGTGCTTTGGGAGTGCTGGTCCAAGGGCGTTAATGGACA-3′ S1 Aptamer-T (5′-Biotin-TTTTTCAGCACCGACCTTGTGCTTTGGGAGTGCTGGTCCAAGGG CGTTAATGGACA-3′) N Aptamer-T (5′-Biotin-TTTTTTGCAATGGTACGGTACTTCCGGATGCGGAAACTGGCTAATTGGTGAGGCTGGGGCGGTCGTGCAGCAAAAGTGCACGCTACTTTGCTAA-3′)	Aptamer binds to the RBD	1 nM–100 nM	0.26 nM	LSPR	[123]
18	SARS-CoV-2	RNA	5′-SH-(A15) CAGCACCGACCTTGTGCTTTGGGAGTGCTGGTCCAAGGGCGTTAATGGACA-3′	Aptamer binds to S protein of target	0.5–8 μg mL^−1^	72 ng mL^−1^	Photoelectrochemical	[124]

**Table 3:** A brief overview of the advantages and limitations of optical and electrochemical aptasensors for virus detection.

Transducer Type	Advantages	Limitations	References
Optical	Real-time detection; reliable, high sensitivity	Sensitive to the surrounding environment; surface modification is one of the main challenges; bulky optical devices required	[114,129]
Electrochemical	Simplicity, miniaturization, low cost real-time detection; the possibility of continuous analysis on different analytes	Need redox elements to enhance the current production; time consuming; sensitive to the surrounding environment	[83,87]

## Data Availability

Not applicable.
